# Variola, or Small-Pox

**Published:** 1838-06-06

**Authors:** N. Chapman

**Affiliations:** Professor of the Theory and Practice of Physic, in the University of Pennsylvania


// THE MEDICAL EXAMINER.

DEVOTED TO MEDICINE, SURGERY, AND THE COLLATERAL SCIENCES.

EDITED BY J. B. BIDDLE, M. D. AND M. CLYMER, M. D.

No. 12.] PHILADELPHIA, WEDNESDAY, JUNE 6, 1838. [Vol. I.

### VARIOLA, OR SMALL-POX.

By N. Chapman, M. D., *Professor of the Theory and Practice of Physic, in the University of Pennsylvania.*

[Continued from page 171.]

It appears from the historical sketch I have presented, that small-pox, most probably, broke out at the siege of Mecca, for the first time, though under what peculiar circumstances it was generated, we have no real information. Conformably, however, to the superstitious spirit of that dark age, there is a miraculous account of its origin, almost too ridiculous to be repeated. The infidels tell us, that, at the moment of their greatest distress, a flock of birds came to their succour, with faces like lions, holding in each claw a small stone of the size of a pea, which, being let fall on the Christian army, occasioned an eruption, by which the whole of it was destroyed, while the latter, not to be outdone, ascribed the calamitous event to an impious stratagem of the devil himself. There is a very old tradition, that it was derived from the camel, and I shall, hereafter, mention some facts calculated to show that it may have pre-existed in horned cattle. Its epizootic origin is, on the whole, I think, deemed the most probable conjecture on the subject, though strong objections may be alleged against it.

That variola now arises from a specific contagion, whatever may have been the cause of its primary development, no one doubts. Equally certain is it, that the virus may exist in the form of palpable matter, or as a subtile emanation, the one operating by contact, the other through the medium of the atmosphere. As regards, however, the distance to which this effluvium may be conveyed, there is not the same certainty. Formerly it was believed that it might be wafted by the winds, and infect, within a great though indefinite space. Of late, a different impression is entertained, and the sphere of its influence in the open air is circumscribed to ten or twelve feet. The experiments of Haygarth, confirmed by those of Ryan, Professor at Lyons, seem to render the point tolerably certain. To me, however, it is not improbable, that the contagious principle is connected with the peculiar odour in the disease, and, as far as this may be smelt, is the danger of infection. An opinion of this sort, universally prevails among common people, and perhaps with adequate grounds.

Whether the contagion can be conveyed by *fomites*, has been lately doubted, at which I am not a little surprised, having thought the affirmative evidence very satisfactory. Medical men engaged in the practice of inoculation and attending patients in all stages of small-pox, not conveying the disease, though adopting no precautions against it, by a change of clothes, or otherwise, is the fact mainly relied on, which surely is inconclusive. More time than such transient visits afford, may be required for the impregnation. The true test would be an exposure to the apparel or bedding of the patient.

Not a little curious, is it, that the contagiousness of the disease, under any circumstances, should so long have escaped detection. No allusion to such a property in it can be traced, till after the time of Sydenham. That close observer, himself unsuspicious of contagion, refers the production of the disease to a peculiar constitution of the season. Boerhaave, however, soon afterwards announced the fact, which has never since been disputed.

It is doubtful, at what period of the disease, this property is acquired; some of the authorities, and, particularly, Heberden and Haygarth, who are among the very highest of them, maintaining that it is not for some days after the appearance of the exantheme, while by others it is alleged that it occurs as early as the preliminary fever, and that the poisonous effluvia escape both from the lungs and the skin. These latter views, however, are without confirmation, as we know that the contagious principle is secreted by the eruption, and have no evidence of its being derived from any other source, it would seem to follow, that it can not exist prior to the formation, and, perhaps, the maturity of the cutaneous affection, though, as in the vaccine, it may possibly be eliminated by the vesicle as well as by the pustule. Nor is it less evident, that since the lungs are destitute of the eruption, they do not concur in the generation or emission of the contagious halitus.

The latent or incubative period of the virus is fourteen days, which, according to my experience, is observed with great regularity. It is for the most part, pretty certain in its effects, and all ages are liable to the disease. Even the fœtus in utero, sometimes, though rarely, becomes infected, and there are several cases recorded by Jenner, of its happening, where the mother entirely escaped, from previous protection. Nevertheless, some individuals, however exposed, have no susceptibility to it, continuing exempt through a long life. It has indeed, been calculated, that one in fifty has such a constitutional immunity. But this estimate seems too large, and, in no instance, is an exemption from it to be too confidently relied on. Examples are numerous, where, after escaping for a term of years, susceptibility has been awakened by some mysterious change of condition, and the disease has attacked, and very often fatally, under such circumstances.

But, while contagion must be admitted as an inherent and uniform quality of small-pox, it is not less certain, that it is materially dependent for its nurture and dissemination, as well as for its infinite modifications, on an epidemic influence. That it ever arises, *de novo*, from the same combination of circumstances, which first called it into existence, the state of our knowledge is not sufficiently precise, to determine positively. But such was the common opinion of the early historians of the disease, and which is not wanting in support from the manner of its occasional subsequent recurrences. It has broken out, from time to time, without our being able to trace its revival to any concealed contagion, spreading most rapidly, widely, and always in its worst shapes, from country to country, exercising an unequivocal sway over all other diseases; then suddenly disappearing, to return again at some future period, in all which features, conforming to the phenomena and laws of epidemics. The late prevalence of it supplies a striking illustration of this fact. Breaking out at Edinburgh, in a few years it pervaded nearly the whole world, exhibiting every where all those traits which I have mentioned. Thus existing, the cause also proves far more efficient, and operative, so much so, indeed, that it subverts, in many instances, the protection afforded by previous attacks, whether naturally or artificially acquired, and secondary small-pox becomes an ordinary event.

From the histories of the disease, we shall find these statements most abundantly confirmed. Nor are we without more immediate evidence of it. In 1823, when small-pox appeared in this city, after a long interval, it could not be traced to any imported or derivative source of contagion; cases sprang up, as it were, spontaneously, at a distance from each other, independent of any probable intercourse, wearing, universally, a most inveterate character, and the failures of vaccination and variolation were numerous, with some few examples of the disease, previously had in the natural way, affording no security. As further proof of the dominant epidemic influence at the time, it may be stated, that in the whole compass of our experience, never was exhibited such a tendency to cutaneous affections, every disease, whatever might be its nature, displaying in its course, some eruptive appearance, and often of the most anomalous character and aspect.

Notwithstanding the plausibility of the conjecture, to which I have alluded, that the contagion of small-pox, under certain circumstances, is occasionally generated anew, I am not disposed to adopt it. To me it seems more probable, that the semina of contagion, like those of plants, or ova of animals, and particularly of insects, may remain dormant for an indefinite period. Exactly as the latter are hatched into existence, by a proper degree of temperature, and other propitious circumstances, so is required, to bring the former into activity, a peculiar constitution of atmosphere. We are not wanting in proof, that the seminal principle, in each of the instances cited, will endure for a long term of years in a latent state, waiting, as it were, for the vivifying impulse to be supplied,—and why may it not be equally true in regard to contagion? The musquito, the locust, not to enumerate more examples, disappear for a protracted season, having deposited their eggs, to be awakened into life, at some favourable conjuncture. Every agriculturist is aware of the reversions of certain plants, at remote and irregular periods, the seeds of which must have remained in the soil. As clearly, does it seem, that the material of this, and all other contagious diseases, is governed by a similar law. Much reliance, I am aware, has been placed on the doctrine of equivocal generation, in the explanation of some of the preceding phenomena. But can it be reasonably credited, that any fortuitous combination of elements, which this doctrine supposes, is productive of such definite results?

It may be finally demanded, if the action of the variolous cause can be suspended for months, by change of season only, why may it not be by other influences for years? Thus, we have seen, among ourselves, the disease raging during winter, and ceasing, on the approach of spring, to appear again, on the return of cold weather, and pursuing this order for a lengthened period. It might, perhaps, be shown, though I am not prepared to extend the doctrine so far, that every disease has one definite cause, which is inert or active, according to the presence or absence of those circumstances by which its operation is controlled. Can we, at least, on any other supposition, so easily explain their occasional prevalence on the reverse, and, particularly, epidemic visitations, and their suspensions? Nearly every disease may assume this character, and its being sporadic or more general, mild or otherwise, I think must be referred to the extent and force of the agency by which the cause of the disease is developed and strengthened.

The variolous eruption is so strongly and unequivocally designated, when well evolved, that it can seldom be mistaken. The case with which it may most probably be confounded, is Varicella, or chicken-pock; and, even here, the discriminating circumstances are, for the most part, prominent, and well defined. The latter is preceded by little febrile or other uneasiness, and about the second day of the attack vesicles appear, which, by the fourth or fifth day, dry away,—whereas, at this time, the eruption of small-pox has not gone through even its earliest stage. The eruption, too, in Varicella, is more apt to come out in successive crops, and is, as I have said, vesicular, and not pustular. The vesicle is pointed, and filled with lymph; while that of small-pox is flat, and indented in the middle, and does not lose these peculiarities till it reaches complete maturity, when, distended with pus, it becomes ovate or globular. Devoid of the cellulated structure of the variolous pustule, the varicellous vesicle is, moreover, merely viscid cuticle, easily ruptured, when, or by puncture, the whole of the fluid at once runs out.

Nevertheless, it cannot be denied, that, in some instances, where chicken-pox is very violent, it assumes so much the aspect of small-pox, as to embarrass practitioners the most conversant with the two diseases. Even Willan, so learned in eruptive affections, confesses this; and, I have known the same degree of perplexity to occur in this city. There is also a disease, the *Varioloid*, that has lately prevailed to a great extent, in which the difficulty of diagnosis is sometimes considerable. Generally, however, it may be recognised by the greater mildness of the prelusive fever, by the earlier appearance of the eruption, which is scanty, and rather vesicular than pustular, by the absence of the variolous odour, and by the more speedy commencement of desiccation,—as well as by its leaving behind, in place of pits, or indentations, a smooth red surface, or small excrescent elevations. As, however, in Varicella, cases of it are not unfrequent, so intimately allied to Variola, in every feature, as to defy the best powers of discrimination.

Little is usually to be apprehended in the discreet or distinct small-pox. The confluent, on the contrary, is always alarming, though less so where the fever is inflammatory, and the vital forces unimpaired. By the aid of common skill, the constitution will work out its preservation.

Never exempt from danger is the typhoid or congestive condition, and, when malignant, scarcely a hope can be indulged. Much may be deduced from some other circumstances. Nearly always fatal is the disease in advanced life, very generally so in the pregnant state, and is most favourable between the second and tenth years of age. The season of puberty, in females, is said to be unpropitious, by a high authority,* which I have not remarked. An eruption, entirely over the surface, though not confluent, is dangerous, from the injury done to the skin, and is particularly so where the face is thickly covered. The character of the pustules, too, materially influences the result. It is best, when they are elevated, round, environed by a definite red areola, and filled with thick yellow pus,—coming out seasonably, and passing regularly through the several stages to maturity. Deviations in any respect from this are calculated to excite solicitude, and especially where the eruption either lingers in its appearance, or comes out prematurely, or the pustules are flat, and nearly empty, or indurated, or filled with a lymphy, or serous, or darkish fluid, and, above all, with blood. The entire cessation of fever, with pallor, and other evidences of collapse, during the eruptive stage, is alarming, and, particularly, if followed by suppression of urine, or recession of the eruption. Either the sudden subsidence of the swelling of the hands, or of the salivation, is a bad indication. Complicated with any of the affections formerly noticed, the danger is considerably enhanced, though less, when inflammatory, than heavily congestive. Laryngitis, however, is an exception, which nearly always proves fatal, on attaining to any height. Great derangement in the cerebral and nervous system, evidenced by tremors, subsultus tendinum, stupor, or delirium, slow and muttering, or wild, particularly the *delirium ferox* of the old writers, is, moreover, of the worst import. Not less so, perhaps, are passive hæmorrhages from the bowels or other parts, or petechiæ, vibices, sloughing of the fauces, and the other signs of what was once considered a putrid diathesis.

The eleventh and thirteenth days are usually the most dangerous, though the eruptive stage throughout is critical, and much is to be apprehended from any extraordinary violence of the secondary fever. But under all circumstances, natural small-pox proves frightfully fatal. It is computed, that one out of four dies, whatever may be the advantage of situation, or the degree of medical skill and attention, and this mortality takes place, when there is no extraordinary violence in the disease. From the reports of the London Small-Pox Hospital, it appears that, for the last fifty years, the deaths have averaged about thirty, though, on some occasions, amounting to forty per cent. Characterized by the malignity incident to some of the epidemic occurrences of it, two out of three perish, or even a greater proportion. During its last prevalence in this city, we lost, in the beginning, more than one-half in hospital practice. The same degree of mortality has happened elsewhere, and, especially, in the hospital at Ceylon, when the disease prevailed there, epidemically, in the year 1819.

(To be continued.)

* Richter.

